# 

**Published:** 1856-12

**Authors:** 


ARTICLE I.—Homœopathy. By ROBERT E. CAMPBELL, M. D., of Benton, Lowndes county, Alabama,........ 193

ARTICLE II.—Diseases of Atlanta, Georgia. By J. G. WESTMORELAND, M. D., Atlanta, Georgia,........ 198

ARTICLE III.—Severe Headache from Carious Teeth,........ 201

ARTICLE IV.—Case of Fever. By THOMAS S. DENNY, M. D., Atlanta, Ga.,.. 202

### *SELECTIONS.*

Annual Address of the President of the Illinois State Medical Society........ 205

On Neuralgia; with the Report of two cases of Excision of the Nerve,........ 215

Reminiscences of the late Dr. Physick, and a few of his contemporaries,........ 222

Influence of the Climate of Peru on Pulmonary Consumption,........ 229

Letter from New York,........ 237

Case of Infantile Epilepsy,........ 233

Medical Society of the State of California,........ 239

### *EDITORIAL AND MISCELLANEOUS.*

Editorial Correspondence,........ 241

Atlanta Medical Society,........ 246

Bibliographical,........ 251

The Sydenham Society of London,........ 253

Medical Miscellanies,........ 254

Etherization in Puerperal Convulsions,........ 255

Mode of Testing the Translucency of Hydrocele,........ 255

Spender's Chalk Ointment in ulcers of the Leg,........ 256

Receipts,........ 256

Errata,........ 256

